# Left-Hemispheric Asymmetry for Object-Based Attention: an ERP Study

**DOI:** 10.3390/brainsci9110315

**Published:** 2019-11-08

**Authors:** Andrea Orlandi, Alice Mado Proverbio

**Affiliations:** Neuro-MI, Milan Center for Neuroscience, Dept. of Psychology, University of Milano - Bicocca, Milan 20126, Italy; mado.proverbio@unimib.it

**Keywords:** selective attention, object-based attention, hemispheric asymmetry, ERP, selection negativity, swLORETA, anterior cingulate cortex, visual recognition

## Abstract

It has been shown that selective attention enhances the activity in visual regions associated with stimulus processing. The left hemisphere seems to have a prominent role when non-spatial attention is directed towards specific stimulus features (e.g., color, spatial frequency). The present electrophysiological study investigated the time course and neural correlates of object-based attention, under the assumption of left-hemispheric asymmetry. Twenty-nine right-handed participants were presented with 3D graphic images representing the shapes of different object categories (wooden dummies, chairs, structures of cubes) which lacked detail. They were instructed to press a button in response to a target stimulus indicated at the beginning of each run. The perception of non-target stimuli elicited a larger anterior N2 component, which was likely associated with motor inhibition. Conversely, target selection resulted in an enhanced selection negativity (SN) response lateralized over the left occipito-temporal regions, followed by a larger centro-parietal P300 response. These potentials were interpreted as indexing attentional selection and categorization processes, respectively. The standardized weighted low-resolution electromagnetic tomography (swLORETA) source reconstruction showed the engagement of a fronto-temporo-limbic network underlying object-based visual attention. Overall, the SN scalp distribution and relative neural generators hinted at a left-hemispheric advantage for non-spatial object-based visual attention.

## 1. Introduction

It is known that visual attention can be consciously directed towards a selected location in space (spatial attention [1,2]), a stimulus as a whole (object-based attention [3,4,5]), or specific features of a stimulus (feature-based attention [6,7,8]). While both behavioral and neural correlates of spatial and feature-related attentional processes have been extensively investigated [9,10,11], attentive selection for objects requires further consideration. For instance, a processing bias (i.e., increased accuracy and faster response), together with the enhanced engagement of fronto-parietal regions (i.e., frontal eye-field, posterior parietal cortex), was reported for stimuli occurring within a target location [12]. Several studies have also shown that attention directed towards single (or combined) stimulus features (i.e., color, orientation, motion, spatial frequency) leads to increased activity within the visual areas sensitive to the target physical trait (i.e., V4 for color; MT+ (middle temporal) for motion [8,13]).

In the case of attention on an object, a few studies have shown an enhanced engagement of the extrastriate visual cortices for target stimulus processing [3]. At the same time, the neural mechanisms and relative brain regions underlying this top-down modulation of the occipito-temporal regions are not yet entirely clarified [4,14]. In two classic fMRI studies by O’Craven and colleagues [3], the participants were presented with stimuli depicting a transparent face superimposed over a house, with one stimulus moving and the other stationary. They were instructed to press a button when a target attribute (i.e., face, house, or motion) was consecutively repeated. Attention directed towards faces, houses, or visual motion resulted in increased activity in the FFA (fusiform face area), PPA (parahippocampal place area), and MT (middle temporal), respectively [15,16,17]. This top-down bias signal for the attended attribute also led to enhanced engagement of the areas associated with the processing of the task-irrelevant attribute. This evidence suggests the occurrence of attentive selection processes for the whole object. Similar results were reported in a subsequent study by Serences and colleagues [4], in which two streams of superimposed faces and houses morphed into the next stimuli at a changing rate of 1/s. Target stimuli required the participants to maintain their attention within the current stream or to shift it toward the other stream. Attention directed to faces and houses led to enhanced activity in the right lateral fusiform gyrus and bilateral medial fusiform gyrus, respectively. Additionally, transient shift-related activity was shown in the right superior frontal sulcus/precentral gyrus and the medial superior parietal lobule, suggesting a role of these regions in non-spatial attentional control processes.

More recently, in a MEG (magnetoencephalography) study by Baldauf and Desimone [14], the participants were shown two streams (spatially overlapping) of faces and houses tagged at different presentation frequencies and instructed to attend to one of them (during target detection). Selective attention enhanced the functional connectivity between the stimulus sensitive visual regions (FFA and PPA) and the inferior frontal junction (IFJ, identified using an attention-related fMRI localizer) at the specific tagging frequencies. Increased phase coherence was also visible in the higher gamma range. The phase lag (20 ms) between frontal and temporal areas indicated the IFJ as a potentially key region for top-down modulation of object-based (and feature-based) attention [18,19]. Using a different approach, Valdes-Sosa and colleagues [20] showed evidence for non-spatial, object-based attention selection at an early stage of vision. The authors presented the participants with two superimposed sets of red and green dots. They were instructed to detect coherent motion displacements (in cardinal directions) of one set of dots, ignoring the other one. The two sets could also be stationary or in rigid rotation around the central fixation point in both the same and opposite directions. In the latter baseline condition (perceived as two transparent superimposed surfaces), unattended (rather than attended) stimuli resulted in a suppression of the posterior N1 and P1 components related to the motion onset. This effect was reduced or absent when the two sets were perceived as a single object, while the attended (than unattended) stimuli elicited an increased selection negativity (SN) response.

Another issue that needs more in-depth analysis relates to the roles of the left and right hemispheres in selective attention processes. A right asymmetry has been shown during tasks involving overt and covert attentional shifting to selected spatial locations in healthy and clinical (i.e., neglect syndrome) populations [21,22,23,24]. A greater engagement of the right hemisphere has also been shown during tasks requiring sustained attention [25,26]. At the same time, evidence from several studies seems to suggest left-lateralized neural substrates underlying focused attentive selection [27,28,29,30,31]. A case in point is the ERP (event-related potential) study by Proverbio and colleagues [30]. The participants were presented with images of familiar objects and animals that were associated or not associated with the prototypical color. They were instructed to recognize either the shape or color of the stimuli, ignoring the other trait. Target stimuli characterized by the prototypical (vs. unassociated) color/shape combination elicited a larger N2 component (or SN) over posterior sites, during the attention to color condition only. This effect was found over the left but not right hemisphere, as also confirmed by the topographic map of voltage distribution computed on the difference wave (associated minus unassociated targets). These results likely showed specific involvement of the left occipito-temporal cortex for conjoined color and shape processing of real objects.

Moreover, Milham and colleagues [28] showed hemispheric differences in attentional control based on the response (vs. non-response) conflict level. The participants were engaged in a Stroop task during fMRI scanning. The incongruent color words could give rise to either an eligible (color in the response set) or an ineligible (color outside the response set) response. Greater engagement of the right fronto-parietal network (i.e., anterior cingulate cortex (ACC), superior, inferior, and middle frontal gyrus, superior parietal lobule) was reported in response to incongruent-eligible (vs. neutral) stimuli. At the same time, both incongruent types of stimuli (relative to neutral stimuli) elicited greater activity in the left hemisphere (i.e., middle frontal cortex, precuneus, and superior parietal lobule), which was likely associated with attentional control at non-response (semantic and phonological) levels.

These findings also appear to be consistent with the left-lateralized brain network reported for local (vs. global) stimulus processing (i.e., Navon stimuli [32,33,34,35,36,37]) and perception of high (vs. low) spatial frequencies [38,39,40,41]. In this vein, Martínez and colleagues [41] reported a larger SN component in response to target (vs. non-target) spatial frequencies (black and white checkboard patterns). The discrimination of high frequencies (5 cpd, cycles per degree) elicited an enhanced SN over the left (compared to the right) hemisphere, while the opposite result was reported for low frequencies (0.8 cpd). In a previous study by Yamaguchi and colleagues [36], the participants were instructed to recognize a target letter (within compound stimuli) at the hierarchical level, indicated by a pre-stimulus cue. The local and global targets elicited an increased N2 response (250–350 ms) over the left and right hemispheres, respectively. The attentional shift elicited by the local (rather than global) cue also resulted in a larger negative response (starting at 240 ms after the onset) at left (vs. right) scalp sites, and vice versa. These findings are in accordance with attentive-selection-related brain asymmetry reported by Fink and colleagues [37]. In that study, greater engagement of the left inferior occipital cortex was found during attention directed to local stimulus features, while globally directed attention activated the right lingual gyrus. Moreover, the number of attentional switches between hierarchical levels (in a divided attention task) co-variated with the activity in the left posterior aspects of the superior temporal gyrus and in the right temporoparietal–occipital junction. This evidence suggests a role of the temporoparietal regions in attentional control for local/global processing, consistent with previous evidence from unilateral brain-damaged patients [42,43]. Finally, partially conflicting evidence was reported in the ERP study by Johannes and colleagues [44]. The authors presented the participants with images representing hierarchically composed non-linguistic stimuli during a divided attention task (detection of a target stimulus at both local and global levels). Target stimuli elicited a larger posterior negative component (Ne, 250–500 ms) which was larger over the left than the right hemisphere for both local and global targets. Global non-targets (distracters) led to an earlier Ne onset during local target processing, suggesting different mechanisms for local/global analysis. At the same time, the asymmetrical distribution on the Ne possibly indicated a predominant role of the left hemisphere in hierarchical stimulus processing.

The present study aimed to further investigate the neural mechanisms underlying object-based visual attention [3,4], under the assumption of a left-hemispheric advantage [28,44]. The EEG (electroencephalography) technique was used, due to its high temporal resolution. Several subprocesses occurring during a target detection task have been previously revealed, indexed by different ERP components modulated (in amplitude and latency) by selective attention. This includes the frontal N2, occipito-temporal N2 (or SN), and centro-parietal P300 responses. These components are interpreted as an index of response inhibition [45], attention allocation [46], and stimulus categorization [47], respectively. Evidence of an earlier effect of visual attention on stimulus processing has also been reported, opening a debate that remains unresolved. While a few authors have claimed an impact of visual attention starting at the level of the extrastriate cortex (i.e., P1 component at 70–75 ms [48]), other authors have reported evidence of a prior modulation of the striate cortex as well (i.e., C1 component at 40–60 ms [49,50]).

Here, 3D graphics were used to create images of three visually comparable (by percentage of non-empty pixels, spatial distribution, etc.) categories of stimuli (wooden dummies, chairs, structures of cubes), which lacked detail. The participants were presented with the stimuli individually displayed at the center of the screen during EEG recordings. At the beginning of each run, they were told which target category required a motor response (button press with the index finger) when perceived between non-target stimuli [51]. Modulation of the amplitude of the N2 (decrease) and P300 (increase) components in response to correctly identified targets (relative to non-targets) was expected over frontal and centro-parietal sites, respectively [45,47]. Moreover, a left asymmetric distribution of the occipito-temporal selection negativity (N2 to target minus non-target) would suggest a predominant role of the left hemisphere in selective visual attention towards objects [30,51]. This hypothesis was also further investigated by applying the standardized weighted low-resolution electromagnetic tomography (swLORETA) inverse solution to estimate the neural sources in the SN time window.

## 2. Materials and Methods

### 2.1. Participants

Twenty-nine healthy, right-handed volunteers took part in the present investigation (13 males and 16 females). They were students at the University of Milano-Bicocca (mean schooling: 16.62 years, SD = 2.03) with an average age of 25 years (mean age: 25.10, SD = 4.96). Each volunteer reported no history of drug abuse or neurological illness, and had normal or corrected-to-normal vision. The right-handedness was assessed using the Italian version of the Edinburgh Handedness Inventory (mean index score: 0.79, SD = 0.17). The experiments were conducted with the understanding and the written consent of each volunteer. The study was conducted in accordance with the Declaration of Helsinki, and the protocol was approved by the Ethics Committee of the University of Milano-Bicocca (protocol number 0000273/14).

### 2.2. Stimuli

**Figure 1:**
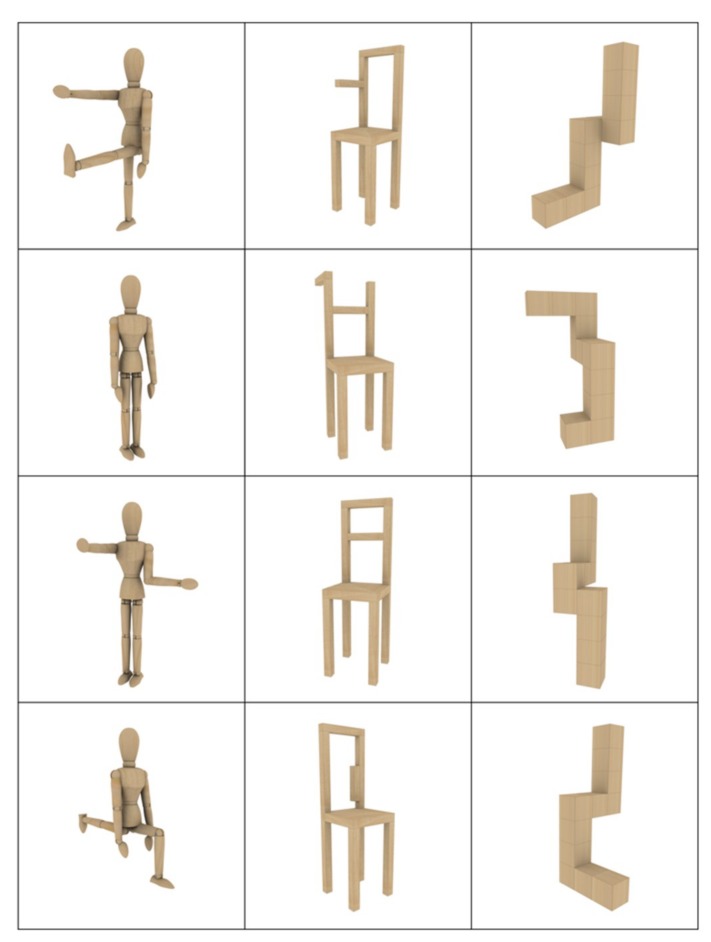
Example of stimuli. The figure shows a few examples of the stimuli used in the present study. Bodies (wooden dummies), objects (chairs), and cube structures were created as 3D graphics. The stimuli set included 240 different images: 16 models for each of the three categories of stimuli, presented from five different points of view obtained by rotating each model along its vertical axis (−40°, −20°, 0°, +20°, +40°).

The stimulus set consisted of 240 different color images created as 3D graphics using Blender software v 2.79. The images depicted wooden dummies (80 stimuli), structures of cubes (80 stimuli), and chairs (80 stimuli) on white backgrounds (see Figure 1). The stimuli were composed of modular structures (cylindrical for the dummies, cubical for the cubes, and rectangular for the chair) which lacked detail (i.e., face, hair, hands/feet in the case of the wooden dummies) and were characterized by a light wood-like texture. This method was illustrated in a previous study by our research group [51]. Presently, 16 models were designed for each of the three stimulus categories (i.e., a mannequin with: both arms at side, left arm extended forwards) and then rotated around the vertical/longitudinal z-axis (0°, ±20°, ±40°). For each model, five points of view were obtained. This increased the visual variety and reduced eventual habituation effects due to stimulus repetition (3 types × 16 models × 5 rotations = 240 stimuli). The stimulus categories were balanced for stimulus distribution in the four quadrants of the visual field. The maximum size of the stimuli was 3.75 × 3.95 cm, subtending visual angles of 1° 53′ × 1° 59′. No difference in stimulus luminance (≈15.9 *cd*/$m^{2}$, *p* = 0.29) and volume occupation (non-empty pixels: ≈10.86%, *p* = 0.39) was revealed by the ANOVAs as a function of stimulus category. Moreover, since each stimulus served as both target and non-target in non-consecutive experimental runs (as illustrated in the following paragraph), there was no difference in mean luminance due to variation in directed attention (target vs. non-target).

### 2.3. Task and Procedure

**Figure 2:**
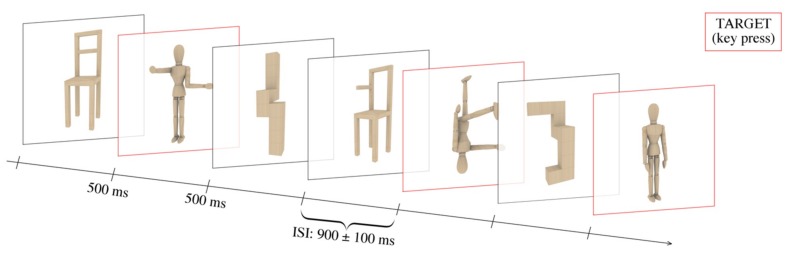
Timescale of the experimental design. The stimuli were presented for 500 ms at the center of the screen, separated by an ISI (inter-stimulus interval) of 900 ± 100 ms. The participants were instructed to recognize a specific target category (i.e., bodies, as illustrated by the red squares in the present figure), indicated at the beginning of each run, by button press.

Once the EEG cap was placed, participants were invited to seat in an acoustically and electrically shielded cabin, facing a high-resolution VGA (video graphics array) computer screen 114 cm away from their eyes. A fixation dot remained at the center of the screen for the entire duration of the experiment. The participants were invited to fixate upon it in order to minimize motion artifacts (i.e., eye gazes, blinks, and body movements) during EEG recording. The stimulus presentation was performed using Eevoke v2.2 (ANT Nneuro, Hengelo, The Netherlands). Each trial consisted of an image centrally displayed for 500 ms and followed by an empty, isoluminant white background for 900 ms ± 100 ms (inter-stimulus interval, ISI). Each of the 240 images was repeated twice during the experiment (in non-consecutive runs) and presented in both upright and upside-down orientations, for a total of 960 stimuli. An experimental run included 80 trials in a pseudo-randomized order, counterbalanced for the type of stimulus (bodies, cubes, chair) and orientation (upright, upside-down). Twelve different runs were created and presented in pseudo-randomized and counterbalanced order to the participants. Before the beginning of the EEG recording, the participants were provided with the experimental instructions (printed and standardized) and engaged in practical training (using two additional runs) to familiarize them with both the task and setting. The participants were asked to identify a specific target stimulus, regardless of the orientation, with a button press on a joypad using the index finger (see Figure 2). The target was verbally indicated by the experimenter at the beginning of each run and represented one-third of the total images displayed in that run. The left and right hand were used alternatively between runs, and the order was counterbalanced across volunteers. All the participants were blinded to the goal of the study and the stimulus proprieties.

### 2.4. EEG Recording and Data Analysis

EEG data were recorded using a standard EEG cap with 128 electrodes located according to the 10–5 International System [52] using EEProbe v2.2 software (ANT Nneuro, Hengelo, The Netherlands). The sampling rate was 512 Hz, and averaged mastoids represented the reference electrode. Electrooculograms (EOG) were also collected. The impedance of the electrodes was maintained below 5 kΩ. The EEGs and EOGs were amplified and subjected to a half-amplitude band-pass filter (0.16–70 Hz, 50 Hz notch). An automated artefact rejection procedure was used to remove EEG segments marked by eye movements (saccades and blinks), muscle-related potentials, or amplifier blockages. Peak-to-peak amplitudes superior to 50 μV were considered artefacts. Trials containing errors (non-targets wrongly indicated as targets) and omissions (wrongly unrecognized targets) were also manually discharged. EEG epochs were synchronized with the stimulus onset. ERPs were averaged, considering −100 ms before the stimulus onset and 1000 ms after the onset. They were subjected to a band-pass filter of 0.16–30 Hz. ERPs were identified and measured with reference to the average baseline voltage, computed as the 100 ms before the stimulus onset. The electrode sites and ERPs’ latency were chosen based on the maximum amplitude reached by the components of interest [53] and in accordance with previous literature [45,46,47]. For the purposes of the present manuscript, only the stimuli presented in the upright orientation were considered (50% of all trials). This avoided any possible confounding effect lead by the inversion of stimuli depicting bodies but not objects [51,54,55]. ERP averages were computed as a function of attention, electrodes, and hemisphere factors. The two levels of the attention factor (target, non-target) were obtained by collapsing all the target and non-target images, respectively, regardless of the content (to increase the EEG signal-noise ratio).

**Figure 3:**
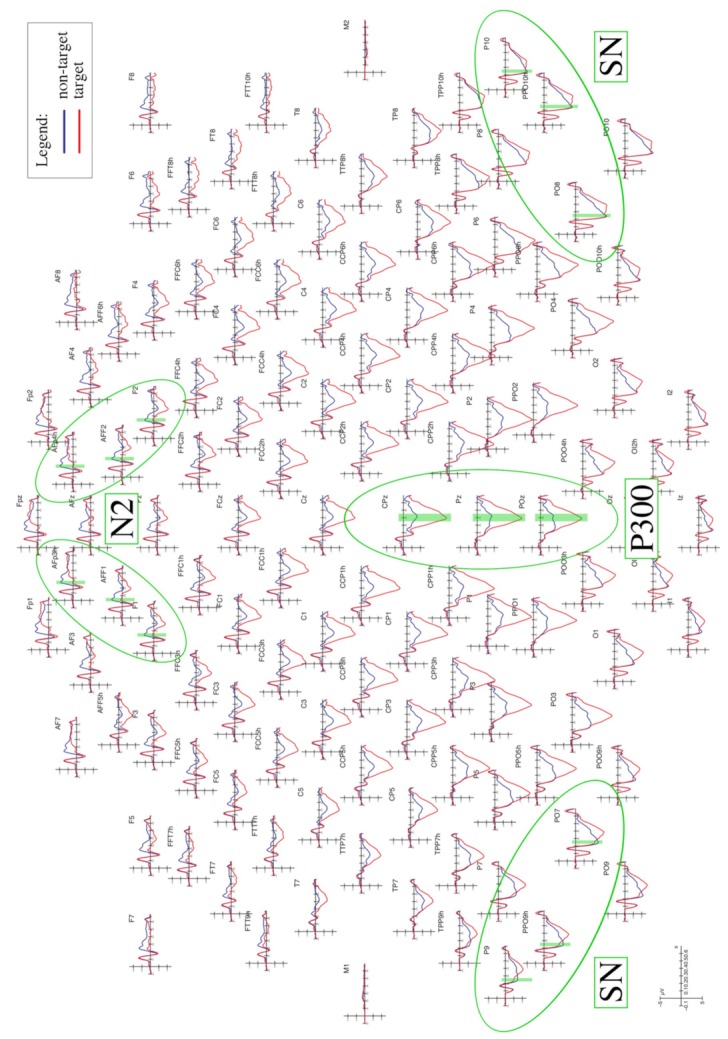
Grand average event-related potential (ERP) waveforms recorded over the scalp. Grand average waveforms (ERPs) recorded over the entire scalp (128 electrodes). The red lines represent the evoked response to target stimuli, while the blue lines represent the evoked response to non-target stimuli. The electrode sites where the three components of interest (N2, SN, and P300) reached the maximum peak are shown by the green circles. The time windows in which the N2 (225–265 ms), SN (240–280 ms), and P300 (350–450 ms) were analyzed are highlighted by the green areas.

The mean area voltage of the N2 component was measured at AFp3h, AFp4h, AFF1, AFF2, F1, and F2 electrode sites during the 225–265 ms time window (see Figure 3). The mean area voltage of the selection negativity (SN) component was measured at P9, P10, PPO9h, PPO10h, PO7, and PO8 electrode sites during the 240–280 ms time window. The mean area voltage of the P300 component was measured at CPz, Pz, and POz electrode sites during the 350–450 ms time window. The N2 and SN data were subjected to multifactorial repeated measures ANOVA with three within-group factors, including: attention (non-target, target), electrode (three levels depending on the ERP component of interest), and hemisphere (left, right). The P300 data were subjected to multifactorial repeated measures ANOVA with two within-group factors, including attention (non-target, target), and electrode (CPz, Pz, POz) factors. Multiple comparisons were computed using Tukey’s post-hoc tests; all the ANOVAs were performed using Statistica software (version 10, Tulsa, OK, USA) by StatSoft.

Standardized weighted low-resolution electromagnetic tomography (swLORETA) was applied to the difference waves obtained by subtracting the ERPs for the non-target stimuli from those elicited by target stimuli in the SN time window (240–280 ms). LORETA, which is a discrete linear solution to the inverse EEG problem, corresponds to the 3D distribution of neuronal electric activity that yields maximum similarity (i.e., maximum synchronization) in terms of orientation and strength between neighboring neuronal populations (represented by adjacent voxels). In this study, an improved version of the sLORETA (standardized low-resolution electromagnetic tomography) was used, which incorporates a singular value decomposition-based lead field weighting (swLORETA) [56]. The following characteristics for source space were included: five points of grid spacing (the distance between two calculation points) and estimated SNR (Signal-to-Noise Ratio defines the regularization; a higher SNR value leads to less regularization and less blurred results) equal to three. The source reconstruction was performed on group data to identify statistically significant active electromagnetic dipoles (*p* < 0.05).

The accuracy (percentage of hits), reaction times (RTs), and errors (percentage of wrong responses to non-targets) were also recorded and measured. Repeated measures ANOVAs were performed on the mean RTs, and percentages of hits and errors with one within-group factor: hand (left, right).

## 3. Results

### 3.1. Behavioral Results

#### 3.1.1. Accuracy

The mean accuracy (HITs) reached the 99.5% level and no difference as a function of handedness was shown (*p* = 0.41). The error rate, intended as the number of non-target stimuli wrongly identified as targets, reached 0.9%, with no difference between the right and left hand (*p* = 0.62).

#### 3.1.2. Reaction Times

The ANOVA performed on the reaction times (RTs) showed an almost significant trend (F(1,28) = 3.697, *p* = 0.06, $\eta_{p}^{2}$ = 0.12) towards a faster response with the right hand (420 ms, SE: 5.96) relative to the left hand (429 ms, SE: 8.15).

### 3.2. Electrophysiological Results

#### 3.2.1. Anterior N2 (225–265 ms)

The ANOVA performed on the amplitude values of the N2 component revealed a significant main effect of attention (F(1,28) = 59.864, *p* < 0.0001, $\eta_{p}^{2}$ = 0.68). The negativity was larger in response to non-target stimuli (−2.24 μV, SE: 0.63) relative to the target stimuli (−0.18 μV, SE: 0.72).

**Figure 4:**
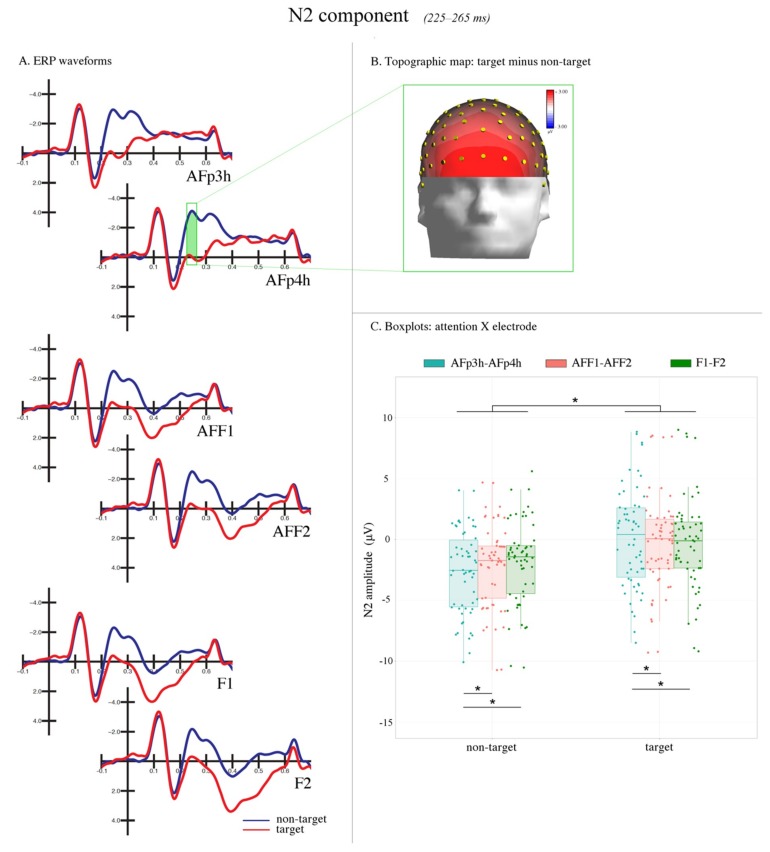
N2 component. (**A**) The grand average ERP waveforms recorded at frontal sites. The correctly recognized targets (in red) relative to non-target stimuli (in blue) led to a reduction of the amplitude of the N2 response between 225–265 ms (area highlighted in green). (**B**) The topographic map (front view) of voltage distribution in the P300 time window (350–450 ms) computed as the difference wave target minus non-target. The positive values are represented in red, while the negative values are represented in blue. (**C**) The boxplots relative to the significant attention X electrode interaction. The N2 response to target (compared with no-target) stimuli was smaller at all electrode sites.

Furthermore, a significant interaction between attention and electrode factors (F(2,56) = 49.215, *p* < 0.0001, $\eta_{p}^{2}$ = 0.64) was confirmed, with a larger frontal N2 elicited by non-target (compared with target) stimuli at all the electrode site considered (*p* < 0.001). Moreover, the N2 measured at AFp3h–AFp4h was significantly different from those measured at AFF1–AFF2 and F1–F2 in response to both target and non-target (*p* < 0.001) stimuli. At the same time, no difference in the N2 amplitude was found between AFF1–AFF2 and F1–F2 (*p* = 0.99) electrode sites. In addition, the N2 recorded at the AFp3h–AFp4h prefrontal sites (non-target minus target: −2.73 μV was more sensitive to the attentive modulation compared with the N2 at AFF1–AFF2 (non-target minus target: −1.85 μV) and F1–F2 (non-target minus target: −1.59 μV) frontal sites (see Figure 4).

#### 3.2.2. Selection Negativity (240–280 ms)

**Figure 5:**
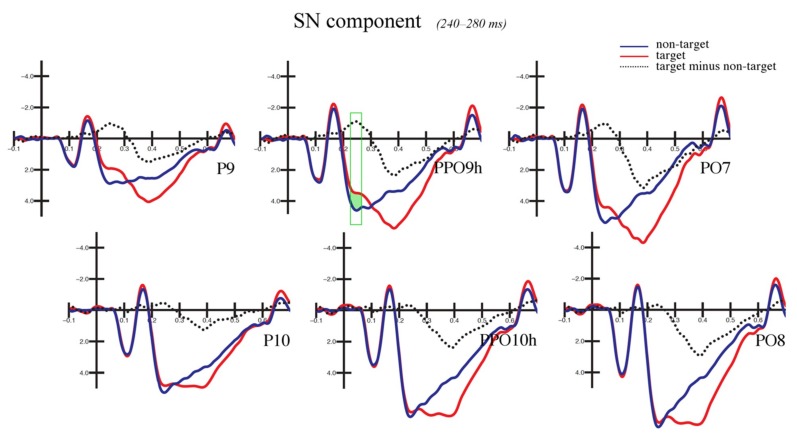
Selection negativity (SN) component: grand average ERP waveforms. The figure illustrates the grand average ERP waveforms recorded at occipito-temporal sites. The correctly recognized targets (in red) relative to non-target stimuli (in blue) led to more negative values of the SN (selection negativity) response between 240–280 ms (area highlighted in green, which also corresponds to the area under the dotted curve). The top row shows electrode sites over the left hemisphere (P9, PPO9h, and PO7), while the bottom row shows those over the right hemisphere (P10, PPO10h, and PO8).

The ANOVA performed on the amplitude values of the occipito-temporal selection negativity (SN) potential showed a significant main effect of the attention factor (F(1,28) = 8.512, *p* < 0.007, $\eta_{p}^{2}$ = 0.23). The SN was more negative in response to target (4.32 μV, SE: 0.51) relative to non-target (4.85 μV, SE: 0.49) stimuli (see Figure 5).

The SN was also larger over the left hemisphere (3.47 μV, SE: 0.46) compared with the right hemisphere (5.70 μV, SE: 0.57), as shown by the significant effect of hemisphere factor on the SN amplitude (F(1,28) = 46.414, *p* < 0.0001, $\eta_{p}^{2}$ = 0.62).

**Figure 6:**
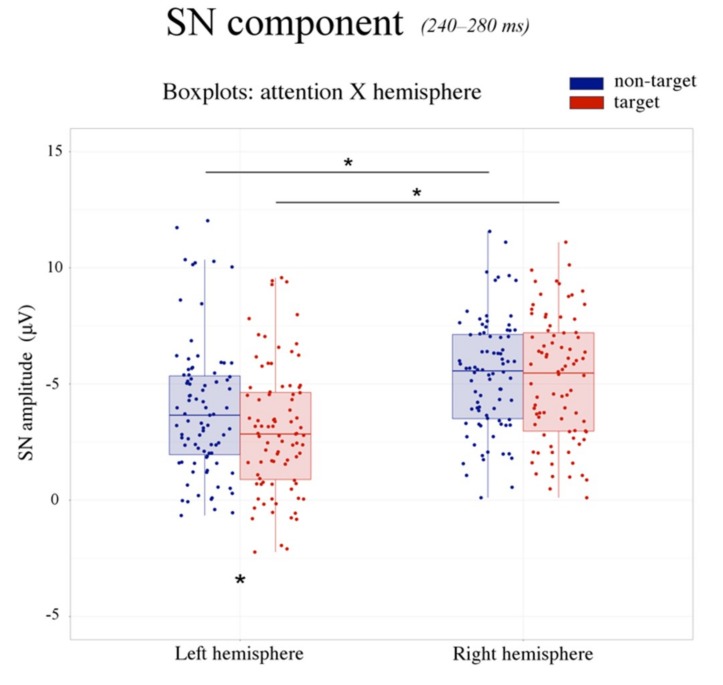
SN component: boxplots. The figure illustrates the boxplots relative to the significant attention by hemisphere interaction for the SN component. The SN was larger over the left than the right hemisphere. Moreover, the SN was more negative in response to the target (in red) compared with non-target (in blue) stimuli over the left, but not the right hemisphere. This evidence suggests that selective attention processes for visual object recognition are left-lateralized.

**Figure 7:**
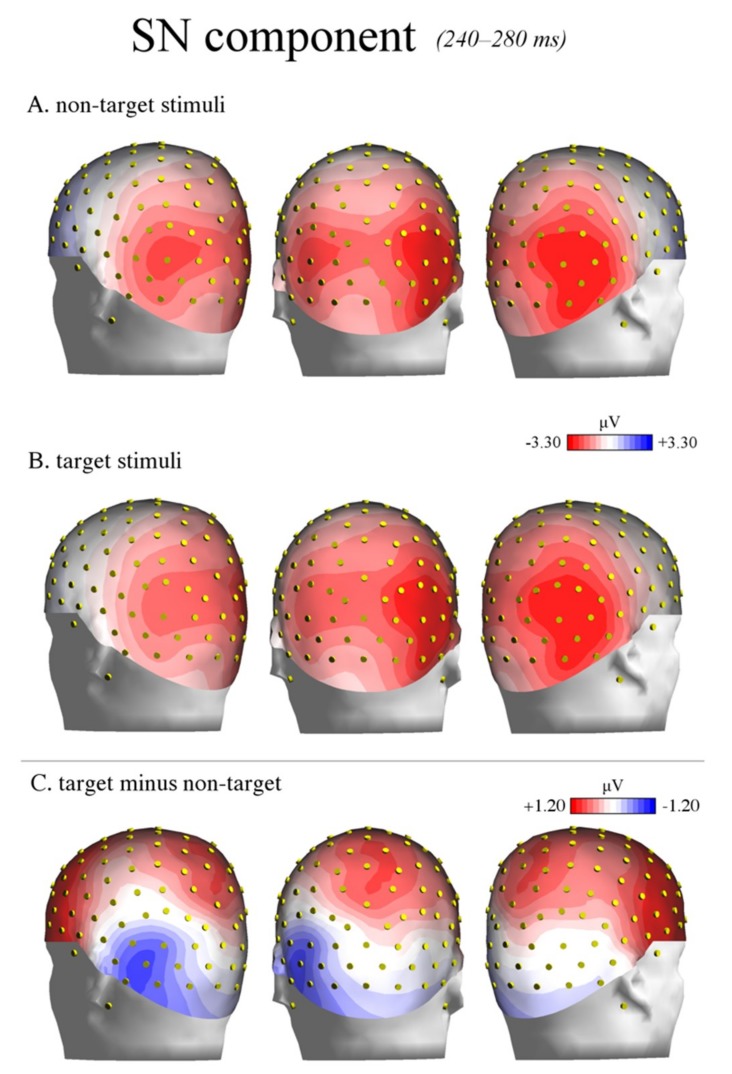
SN component: topographic maps. The figure illustrates the back view of the topographic maps of voltage distribution in the SN time window (240–280 ms) relative to non-target stimuli (**A**), target stimuli (**B**), and the difference wave target minus non-target (**C**). The positive values are represented in red, while the negative values are represented in blue. A strong left-lateralized negative peak is visible at occipito-temporal sites (**C**).

Moreover, the significant attention by hemisphere interaction (F(1,28) = 16.756, *p* < 0.001, $\eta_{p}^{2}$ = 0.37) revealed a more negative SN response to target (relative to non-target) stimuli over the left hemisphere (non-target: 3.92 μV, SE: 0.48; target: 3.02 μV, SE: 0.45; *p* < 0.0002), but not the right hemisphere (non-target: 5.78 μV, SE: 0.55; target: 5.62 μV, SE: 0.61; *p* = 0.64) (see Figure 6 and Figure 7).

Finally, the main effect of the electrode factor (F(2,56) = 67.7041, *p* < 0.0001, $\eta_{p}^{2}$ = 0.71) showed that the SN amplitude maximally peaked at P9–P10 electrode sites (3.38 μV, SE: 0.49) and gradually reduced at PPO9h–PPO10h (4.86 μV, SE: 0.50) and PO7–PO8 (5.52 μV, SE: 0.52), respectively.

#### 3.2.3. P300 (350–450 ms)

**Figure 8:**
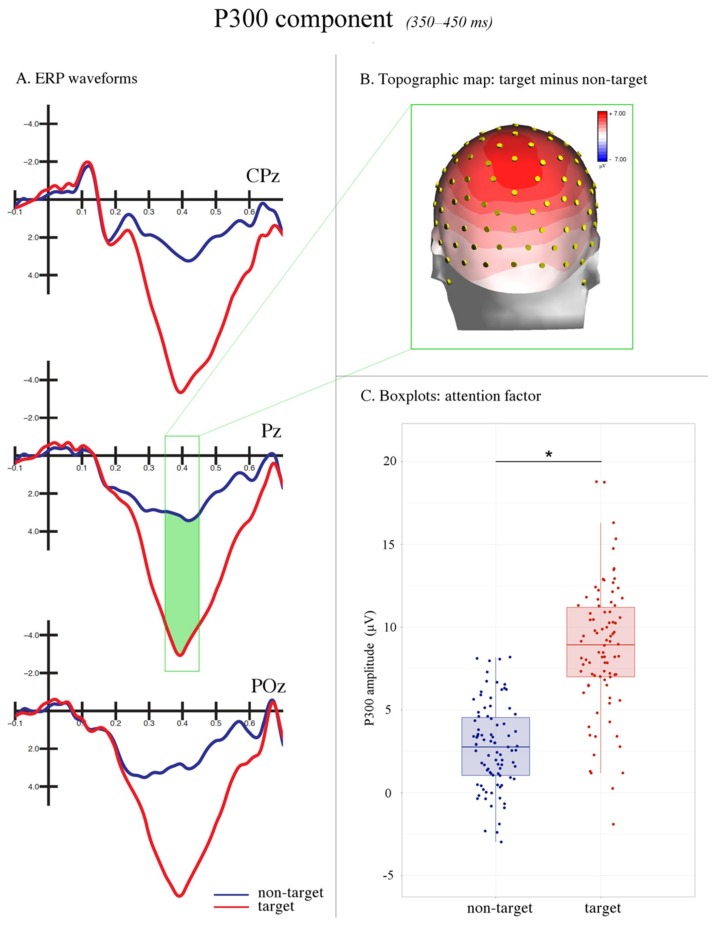
P300 component. (**A**) The grand average ERP waveforms recorded at centro-parietal midline sites. The correctly recognized targets (in red) relative to non-target stimuli (in blue) elicited a larger P300 response between 350–450 ms (area highlighted in green). (**B**) The topographic map (back view) of voltage distribution in the P300 time window (350–450 ms) computed as the difference wave target minus non-target. The positive values are represented in red, while the negative values are represented in blue. (**C**) The boxplots relative to the significant main effect of the attention factor.

The ANOVA performed on the amplitude values of the P300 component showed a significant main effect of the attention factor (F(1,28) = 97.756, *p* < 0.0001, $\eta_{p}^{2}$ = 0.78). The positivity evoked by the target stimuli (8.95 μV, SE: 0.77) was larger than that evoked by non-target stimuli (2.78 μV, SE: 0.46), as can be seen in Figure 8.

#### 3.2.4. swLORETA Source Reconstruction (240–280 ms)

**Figure 9:**
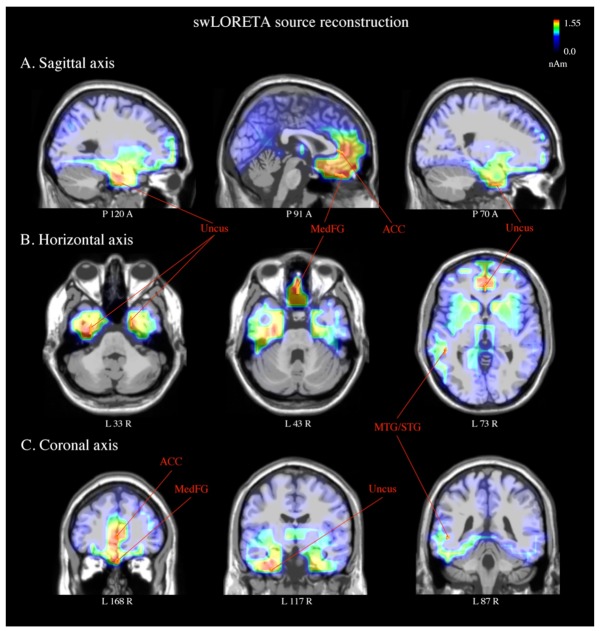
Standardized weighted low-resolution electromagnetic tomography (swLORETA) source reconstruction of surface potentials in the SN time windows. SwLORETA performed on the grand-average waveforms of the difference wave (target minus non-target) in the time window of the SN component (240–280 ms). The sagittal (**A**), horizontal (**B**), and coronal (**C**) anatomical planes of the brain are shown. The engagement of limbic regions is visible, which includes the right anterior cingulate cortex (ACC, BA 24), medial frontal gyrus (MedFG, BA 11), and uncus, bilaterally (BA 28/36). Active dipoles in the left middle/superior temporal gyrus (MTG/STG, BA 22) and inferior frontal/precentral gyrus (IFG, BA 9/6) are also shown. The strongest magnitude values of the signal (nAm) are presented in red.

The swLORETA inverse solution investigated the cortical sources of the bioelectrical activity recorded over the scalp underlying selective attention processes for object recognition. For this purpose, the source reconstruction was applied to the difference wave obtained by subtracting the ERP evoked by non-target stimuli from those elicited by target stimuli in the SN time window (240–280 ms). A list of the estimated active electromagnetic dipoles can be found in Table 1. The main dipoles were located in the medial frontal gyrus (BA 11) and right anterior cingulate cortex (BA 24). The uncus (BA 28/36) was also bilaterally engaged, together with the left middle/superior temporal (BA 22) and inferior frontal/precentral (BA 6/9) gyri (see Figure 9).

## 4. Discussion

The present ERP study investigated the time course and neural correlates of object-based attention, under the assumption of left-hemispheric dominance. For this purpose, healthy, right-handed participants were presented with 3D graphic images depicting the shapes of different categories of stimuli (wooden dummies, chairs, structures of cubes) which lacked detail. They were instructed to pay attention to and detect one given target category (singularly and centrally presented) among non-targets by emitting a motor response (button press). As visible in Figure 3, three main ERP components (N2, SN, and P300) were shown to be sensitive to selective attention in different time windows, likely highlighting different the cognitive processes involved in recognition of the target objects.

The anterior N2 was the first potential that was modulated by the attentive selection (225–265 ms). The amplitude of this negativity was reduced in response to the target images when compared with the non-targets. Similar results have been previously reported during go/no-go tasks. A larger N2 has been found for non-target (relative to target) item tasks that require response inhibition (no-go) in terms of both actual [45] and imagined [57] motor acts (i.e., button press). This interpretation is supported by source reconstruction studies that have localized the neural generators of the N2 in the anterior cingulate cortex (ACC [58]). The ACC has been associated with cognitive control, as shown by several imaging investigations in both healthy and clinical (i.e., Huntington’s disease) individuals [59,60]. The N2 has also been proposed as an index of conflict monitoring [61] and is modulated by stimulus novelty [62], category [63,64], and mismatch [65]. In the present study, the identification of non-target images, which represented two-thirds of the stimuli, required no actual finger movements. Hence, the relative decrease in the N2 amplitude can be considered a correlate of motor inhibition [66]. For instance, in the study by Proverbio and colleagues [66], the participants were presented with four gratings of different spatial frequencies briefly displayed in the four quadrants of the visual field. They were instructed to respond to a target combination of spatial frequency and space location. When compared with the non-targets, the pseudo-targets (stimuli close in spatial frequency to the target and falling within the attended quadrant) elicited both a larger frontal motor N2 and a larger negative prefrontal potential (370–430 ms). This evidence was interpreted as an index of the response inhibition and top-down cognitive control required for irrelevant information suppression. Finally, no hemispheric difference was found here at this stage of stimulus processing (N2 time window). This result is consistent with the increased power in the theta frequency band that has been previously reported at midline frontal scalp sites during target detection tasks [67]. The medial prefrontal cortex has been proposed as a possible neural generator of this effect (i.e., ACC [68]).

Moving forward at the temporal level, the analyses of selection negativity (or posterior N2) response (240–280 ms) revealed an increased amplitude over occipito-temporal areas elicited by target relative to non-target stimuli. This effect is illustrated in Figure 5, which reports the grand average ERP waveforms recorded over posterior sites. The SN was typically obtained subtracting the posterior N2 elicited by non-target stimuli from that evoked by target stimuli. It is considered an index of visual attentive selection processes [46], as it shows sensitivity to several target stimulus features (or a combination of them), including color [69,70], orientation [71], spatial frequency [41,72], and shape [73]. Thus, in the present study, the modulation of the SN may have indicated non-spatial attention allocation towards the specific stimulus shape required for object recognition, consistent with previous evidence [74]. Furthermore, the maximum amplitude of the SN was reached over occipito-temporal scalp sites. The location was compatible with the modulation of associative visual cortex previously reported in several imaging studies on non-spatial attention [3,4].

More importantly, the modulation of the SN was specifically visible over the left but not the right hemisphere, as shown by the topographic maps of voltage distribution depicted in Figure 7 (see also Figure 6). This evidence is consistent with previous ERP studies on shape [51] and color detection [30], as well as local (vs. global) stimulus information processing [32,41] and illusory contour perception [31]. For instance, in a previous study by our research group [51], the participants were presented with images representing upright and inverted bodies (wooden dummies) and structures of cubes. They were instructed to attend to one of the two categories of stimuli (by button press), regardless of the orientation. The occipito-temporal SN was shown to be sensitive to the orientation of the human body shape, being larger in response to the inverted (than upright) body targets. This result likely indicated increased attentive processes for body recognition when presented in non-standard orientations. The negative response was also overall larger over the left than the right hemisphere. This evidence possibly suggests a predominant role of the left hemisphere in shape-related attentive selection processes. It also extends previous findings [30] on conjoined color and shape processing. These results are also consistent with those reported by Zani and Proverbio [72] in their attentional task (relative to spatial frequency). In that study, the relevant/target (relative to the irrelevant) stimuli elicited larger ERP components at both occipital (N165 and P3b) and frontal (LP, long latency positivity) scalp sites over the left but not the right hemisphere. In another study, larger negativity (N2) was elicited by the perception of illusory contours of a Kanizsa square over the left occipital regions [31], consistent with the idea of left-sided non-spatial feature selection and local (vs. global) stimulus processing [36,37].

Furthermore, the swLORETA inverse solution was applied in this study to the difference wave target minus non-target to estimate the neural sources of the EEG signals in the SN time window (240–280 ms). Several active dipoles underpinned the left-sided SN within a fronto-temporo-limbic network, associated with attentive selection processes. This included the ACC (BA 24), the right medial prefrontal cortex (mPFC, BA 11), and uncus, bilaterally (BA 28/36). Several pieces of evidence have linked the ACC and mPFC to cognitive control [75], with the former region specifically involved in performance and conflict monitoring [60,76]. Both the mPFC and uncus (included in the parahippocampal cortices) are part of the affective system of the human brain [77]. Thus, their engagement may also suggest an affective response [78,79] to target stimuli that were correctly identified. More importantly, the swLORETA showed a selective engagement of the superior/middle temporal (STG/MTG, BA 22) and inferior frontal/precentral gyrus (aka IFJ, BA 6/9) in the left hemisphere. A negative correlation between activity in the left STS and response variability has been reported during the perception of oddball (vs. standard) stimuli in healthy (vs. ADHD) volunteers [80]. At the same time, participants with attentional disorders have shown reduced response to oddball (vs. standard) stimuli in the superior and medial temporal lobe (along with the insula and basal ganglia). The STG/MTG is also considered a key node of an amodal semantic hub, together with the temporal poles [81]. Its involvement may be explained in terms of accessing the knowledge (i.e., name [82]) of the target stimulus category (i.e., bodies, chairs). Finally, previous evidence indicated a role of the IFJ (inferior frontal junction) in the top-down modulation of the inferior temporal cortices when a target object was attended [14,19]. The engagement of left frontal regions (i.e., BA 6) has also been reported during executive (conflict) control, together with the ACC [83]. Overall, these findings seem to support the hypothesis of effective engagement of the left hemisphere in selective attentional processes required by feature recognition (i.e., shape) for objects centrally presented in the visual field [4,27,64,84].

Lastly, the effects of attention were also visible at later latencies (350–450 ms) over centro-parietal sites. An increased positive (P300) response to target stimuli was found when compared with non-target stimuli, which likely suggests the recognition of the target object. The P300 response is generally interpreted as an index of item categorization, updating of the mental representation of stimulus context, and visual awareness [47,85,86,87]. The P300 is typically maximal in response to stimuli that are identical to a target. Its neural generators have been estimated within the parietal (i.e., inferior parietal lobule, posterior parietal cortex) and inferior temporal regions [85]. At the same time, non-target stimuli that share some visual features with the target stimulus elicit a gradient of increasing P300s as a function of enhanced similarity [88]. Moreover, the maximum peak of this ERP component is concurrent with the RTs when an accurate but fast response is required. In our study, the average RT to targets was 425 ms, included within the P300 time window considered (350–450 ms). A strong tendency towards a faster response with the right (compared with the left) hand was also found. Previous evidence has shown that simple RTs are not often affected by the hand dominance [89]. This result is also consistent with the hypothesis of left-hemispheric dominance for action selection [90,91]. Hence, the right-handedness of the participants can only partially account for the right-hand advantage reported here. It is important to report the case of target detection during spatial attention modulation. Faster RTs have been found for stimuli occurring in the visual field ipsilateral (relative to the contralateral) to the response hand [92,93,94]. This difference in RTs has been ascribed to the interhemispheric transfer time through the corpus callosum. In the present study, the faster button press obtained with the right hand may have suggested a faster intrahemispheric (vs. interhemispheric) transfer between the contralateral (left) motor cortex and attention-related areas within the left hemisphere. This interpretation, which certainly requires further investigation, is consistent with the assumption of a left-hemispheric dominance in attentive processes for object selection.

A few aspects need further consideration and suggest circumspection in the interpretation of the present results. Firstly, our experimental design likely prevented overt spatial attentional shifting (i.e., central stimuli presentation, stimuli with equal dimensions and number of non-empty pixels, and comparable distribution in the four hemi-quadrants of the visual field), and EEG epochs reporting eye movement were discharged. However, it is also true that covert attentional shifting may have occurred. The introduction of a secondary task would be useful in order to entirely disentangle the contribution of spatial and non-spatial attention [95,96]. This would also strengthen our object-based interpretation of the attention-related results. Secondly, despite a clear left-lateralization of the SN component in our experimental group, individual differences between participants may exist. It is, therefore, desirable for future investigations to account for such variability. Finally, it is necessary to point out that the EEG technique is characterized by a non-ideal spatial resolution [97] compared to functional neuroimaging (i.e., fMRI). This issue can be partially overcome using by high-density caps and state-of-the-art source reconstruction algorithms [98]. Many studies have shown good reliability of swLORETA, since the estimated dipoles were consistent with brain activity found in previous fMRI investigations [99,100]. Caution is still advised when reconstructed neural sources are considered, even when they are in support of main ERP findings, as was the case for the present study.

## 5. Conclusions

In conclusion, these pieces of evidence seem to support the models of selective attention focused on objects as a whole. In our study, the recognition of the shape of a target object that lacked detail modulated several ERP components associated with motor inhibition (N2), attentive selection (SN), and item categorization (P300) processes at different time latencies and scalp distribution. A fronto-temporo-limbic network seems to underlie such object-based attentive processes. The scalp distribution of the SN component and related neural sources (i.e., left MTG/STG and IFJ) were consistent with the hypothesis of a left-hemispheric advantage for non-spatial visual attention. The present results integrate the previous literature on brain asymmetries relative to attentional control for local and global levels of stimulus processing. They seem to suggest a specific role of the left hemisphere in attentive selection and, thus, recognition of objects and relative features.

## Figures and Tables

**Table 1:** List of estimated electromagnetic dipoles.

Magnitude	T-x (mm)	T-y (mm)	T-z (mm)	Hem	Lobe	Gyrus	BA	Function
18.2	1.5	38.2	−17.9	R	F	MedFG	11	Attentive selection
15.3	1.5	35.3	5.3	R	Lim	ACC	24	
5.5	−38.5	2.4	29.4	L	F	IFG/PrecGyrus	6/9	
16.0	−28.5	−8	−28.9	L	Lim	Uncus	28	Affective response
15.8	21.2	−0.6	−28.2	R	Lim	Uncus	36	
12.8	−48.5	−36.6	−1.3	L	T	MTG/STG	22	

List of the electromagnetic dipoles estimated in response to target minus non-target stimuli in the SN time window (240–280 ms) according to swLORETA, with the relative Talairach coordinates. (Legend: Hem—hemisphere, MedFG—medial frontal gyrus, ACC—anterior cingulate cortex, IFG—inferior frontal/precentral gyrus, MTG/STG—middle/superior temporal gyrus, T—temporal lobe, F—frontal lobe, Lim—limbic system, BA—Brodmann’s area, R—right, L—left).
